# LncRNA SNHG12 contributes proliferation, invasion and epithelial–mesenchymal transition of pancreatic cancer cells by absorbing miRNA-320b

**DOI:** 10.1042/BSR20200805

**Published:** 2020-06-05

**Authors:** Wei Cao, Guoxiong Zhou

**Affiliations:** Department of Gastroenterology, Affiliated Hospital of Nantong University, Nantong 226021, Jiangsu, P.R. China

**Keywords:** epithelial-mesenchymal transition, invasion, lncRNA SNHG12, pancreatic cancer

## Abstract

Pancreatic cancer is a kind of malignant carcinoma with high mortality, which is devoid of early diagnostic biomarker and effective therapeutic methods. Recently, long non-coding RNAs (lncRNAs) have been reported as a crucial role in regulating the development of various kinds of tumors. Here, we found lncRNA small nuclear RNA host gene 12 (SNHG12) is highly expressed in pancreatic cancer tissues and cell lines through qRT-PCR, which suggested that SNHG12 possibly accelerates the progression of pancreatic cancer. Further study revealed that SNHG12 promoted cancer cells growth and invasion via absorbing miR-320b. Flow cytometry and transwell chamber assay were utilized to verify the promoting effects on proliferation and invasion that SNHG12 acts in pancreatic cancer cells. Evidence that SNHG12 increased cell invasive ability through up-regulated EMT process was lately obtained by Western blotting assay. Consequently, we extrapolated that SNHG12/miR-320b could be invoked as a promising early diagnostic hallmark and therapeutic strategy for pancreatic cancer.

## Introduction

Pancreatic cancer, a digestive system malignant tumor with high immunosuppression, is in low susceptible level to traditional radiotherapy and chemotherapy. By 2030, pancreatic cancer might become the second most lethal cancer in the United States [1]. Tumor metastasis to peripheral and distant tissues will occur in the development of pancreatic cancer, which resulted in a minimal resection rate of only 20% [2]. With the continuous study on pancreatic cancer, people have discovered that blood vessels and lymphatic metastases exist in the early stages of pancreatic cancer, which was definitely a hindrance in therapy [3]. Blindly pursuing radical surgery or even expanding radical surgery did not improve the prognosis of patients [4]. Roughly 80% of patients have lost their best opportunity to receive surgical treatment when they went to hospital, because of the deficient diagnostic methods and inconspicuous specific clinical syndromes, and the overall 5-year survival rate is less than 6% [5]. Therefore, there is an urgency to identify novel biomarkers for early diagnosis and potential therapeutic strategy to against malignancy progression so as to improve pancreatic cancer prognosis.

Long non-coding RNA (lncRNA), with a length of more than 200 bp, is a kind of transcripts that most exist in the nucleus without protein coding ability [6]. Current investigations suggested that lncRNA could induce and bind proteins involved in transcription, prevent protein complexes from locating in DNA sequences, regulate downstream gene transcription, thus mediates gene expression at multiple levels that make it a crucial factor in promoting or inhibiting tumor progression [7,8]. Recent studies have reported multiple lncRNA imbalance in pancreatic cancer and its metastases may participate in the progression of pancreatic cancer through various pathways [9]. It is also pointed out that lncRNA is a pivotal role in the EMT process of pancreatic cancer through a variety of regulatory ways. For example, Shen et al. [10] explained in their work that lncRNA Xist can regulate EMT by adsorbing miR-429 to up regulate ZEB1, thus promoting the invasion and migration of pancreatic cancer. Hence, further study of the exact expression level and regulatory role of lncRNA in pancreatic cancer will be of great help to promote the development of biomarkers for early diagnosis and treatment of pancreatic cancer.

Small nuclear RNA host gene 12 (SNHG12) is a lncRNA located on chromosome 1 with a length of 675 nucleotides [11]. Previous studies have shown SNHG12 plays an important role in the development of triple-negative breast cancer, prostate cancer, colon cancer, gastric cancer and gliomas. The proliferation, metastasis and invasion of tumor cells are significantly proportional to the expression levels of SNHG12 [11–15]. In the present study, we investigated the pathological association between lncRNA SNHG12 and pancreatic cancer, and disclosed its role in regulating tumor progression which encompassed proliferation, migration and invasion. We also studied the expression of SNHG12 in pancreatic cancer and its correlation with clinicopathological features of patients with pancreatic cancer. This research extrapolated that SNHG12 was a pivotal element in accelerating the progression of pancreatic cancer through augmenting proliferation, increasing migration and invasion.

## Materials and methods

### Clinical samples and cell culture

A total of 15 pairs of pancreatic tissues and adjacent normal tissues, 15 groups of non-metastatic pancreatic cancer tissues and 15 groups of metastatic pancreatic cancer tissues from patients in Affiliated Hospital of Nantong University from May 2015 to May 2018 were resected then in liquid nitrogen cryopreservation, and −80°C for long-time preservation. All human participants related experiments were conducted in agreement with the ethical standards of the ethics committee of the Affiliated Hospital of Nantong University (Approval No.20170227-006) as well as the 1964 Helsinki Declaration. All involved patients have sighed the informed consent documents before the commencement of the study. Human normal pancreatic cell line (HPDE6) and pancreatic cell lines (BXPC3, CAPAN1, PANC1, SW1990) were incubated using DMEM medium (HPDE6, CAPAN1, PANC1), RPMI-1640 medium (BXPC3) or L15 (SW1990) medium containing 10% fetal bovine serum and penicillin (100 U/ml) at 37°C with 5% CO_2_.

### Total RNA extraction of tissues and cells

Pancreatic cancer cells (5 × 10^6^/ml) were collected and tissues (50 mg) were ground using fluid nitrogen, 1 ml of TRIzol was then added to lyse cells and preserve the integrity of RNA simultaneously. After 5-min maintenance at 25°C, 200 μl of chloroform were added and mixed for another 15-min maintenance at 25°C. The mixed solution was centrifugatded at 12,000 rpm under 4°C for 15 min. About 0.5 ml of isopropyl alcohol was added to the separated supernatant and maintained at room temperature for 15 min, then centrifugated to obtain the RNA sediment. RNA extraction was dried and stored under −80°C till experiment.

### Real-time quantitative reverse transcription-PCR analysis

Real-time quantitative reverse transcription polymerase chain reaction (qRT-PCR) analysis of SNHG12 and miR-320b expression was performed through PrimeScript RT reagent Kit and SYBR Prime Script RT-PCR Kits under manufacturer’s instructions. Content of SNHG12 and miR-320b was calculated using 2^−ΔΔCt^ method [16]. The expression of SNHG12 was significantly corelated to GAPDH, and miR-320b expression level was proportional to U6. The following sequences in Table 1 are the primers that used in the experiments:

**Table 1:** Primer sequences used in this research

Primer	Forward sequence	Reverse sequence
SNHG12	5′-TCTGGTGATCGAGGACTTCC-3′	5′-ACCTCCTCAGTATCACACACT-3′
GAPDH	5′-GCACCGTCAAGGCTGAGAAC-3′	5′-TGGTGAAGACGCCAGTGGA-3′
miR-320b	5′-GATGCTGAAAAGCTGGGTTG-3′	5′-TATGGTTGTTCTGCTCTCTGTCTC-3′
U6	5′-GCTTCGGCAGCACATATACTAAAAT-3′	5′-CGCTTCACGAATTTGCGTGTCAT -3′

### Cell transfection

SNHG12 siRNA (si-SNHG12) and negative control siRNA (si-NC), the pLV retrovirus vector for SNHG12 (pLV-SNHG12) or negative control (pLV-NC), miR-320b mimics were transfected into pancreatic cell line CAPAN1 utilizing Lipofectamine 2000 according to the protocol.

### Cell proliferation capacity evaluated by MTT assay

Pancreatic cell lines were seeded into 96-well plates with 5 × 10^3^ cells per well containing 100 μl medium and incubated under 37°C, 5% CO_2_ for 24, 48 and 72 h. Then, 20 μl of MTT reagent per well were added for another 4-h incubation. Add 150 μl of DMSO into each well to make the crystal fully dissolved and the aborbance 570 nm (OD570) was measured by enzyme analyzer within 10 min.

### Cell invasion capacity evaluated by transwell assay

To evaluate the capacity of cell migration, 200 μl of cell suspension (1 × 10^5^ cells) was transferred into the upper compartment of a Transwell chamber (Corning, Corning, NY, U.S.A.), which has an 8 μm pore size and a 24-well insert. In the upper chamber of each well, 50 μl of serum-free medium containing 10 g/l bovine serum albumin was added. All the lower chambers were loaded with 10% FBS. Cell migration capacity was assessed by the number of cells reaching the lower chamber. For invasion assays, the upper chamber was enfolded with Matrigel (BD Biosciences, San Jose, CA, U.S.A.). The other procedures were same as the migration assays.

### Immunoblotting test

Protein isolation and immunoblotting test were exerted as previously reported. Antibodies against SNHG12, miR-320b, E-cadherin, N-cadherin, Vimentin and GAPDH were used, while GAPDH was considered as the internal control.

### Apoptosis level evaluated by flow cytometry

Flow cytometry was exerted to study the effects on cell cycle and apoptosis after transfections. Annexin V-FITC Apoptosis Detection Kit and Cell Cycle Detection Kit were supplied by KeyGEN BioTECH (Nanjing, China). Cells were processed based on the manufacture’s protocol.

### Dual luciferase assay

pGL3 vector and the synthetic SNHG12 containing wild-type (WT) or mutated (Mut) region were utilized to construct the reporter plasmids, which were then co-transfected into cells with miR-320b mimic or miR-NC (miRNA negative control) using Lipofectamine 2000 under manufacturer’s instruction, and incubated for 24 h. Then, luciferase activities of Renilla and Firefly were examined by Dual-Luciferase Reporter Assay System and luminometer.

### Statistical analysis

The results data were expressed as mean of triplicate measurements plus standard deviation (SD). A two-tailed Student’s *t* test was employed to analyze differences between two groups. Multiple comparisons between groups were performed using analysis of variance (ANOVA) followed by Tukey’s post hoc test using SPSS (13.0) or Graphpad Prism 7.0. *P*<0.05 means statistically significant.

## Results

### SNHG12 was in high-expression level in pancreatic cancer tissues and cell lines

**Figure 1:**
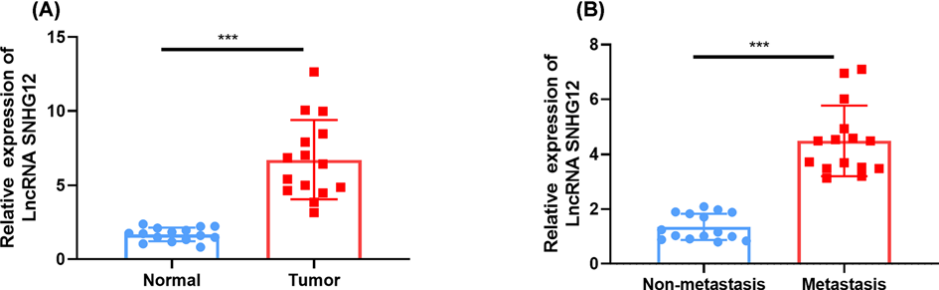
Up-regulation of SNHG12 in pancreatic cancer tissues (**A**) SNHG12 was up-regulated in pancreatic cancer tissues versus normal tissues, which were determined by qRT-PCR. (**B**) Metastatic pancreatic cancer tissues had higher expression level of SNHG12 than non-metastasis. ***P<0.001 vs. respective control.

**Figure 2:**
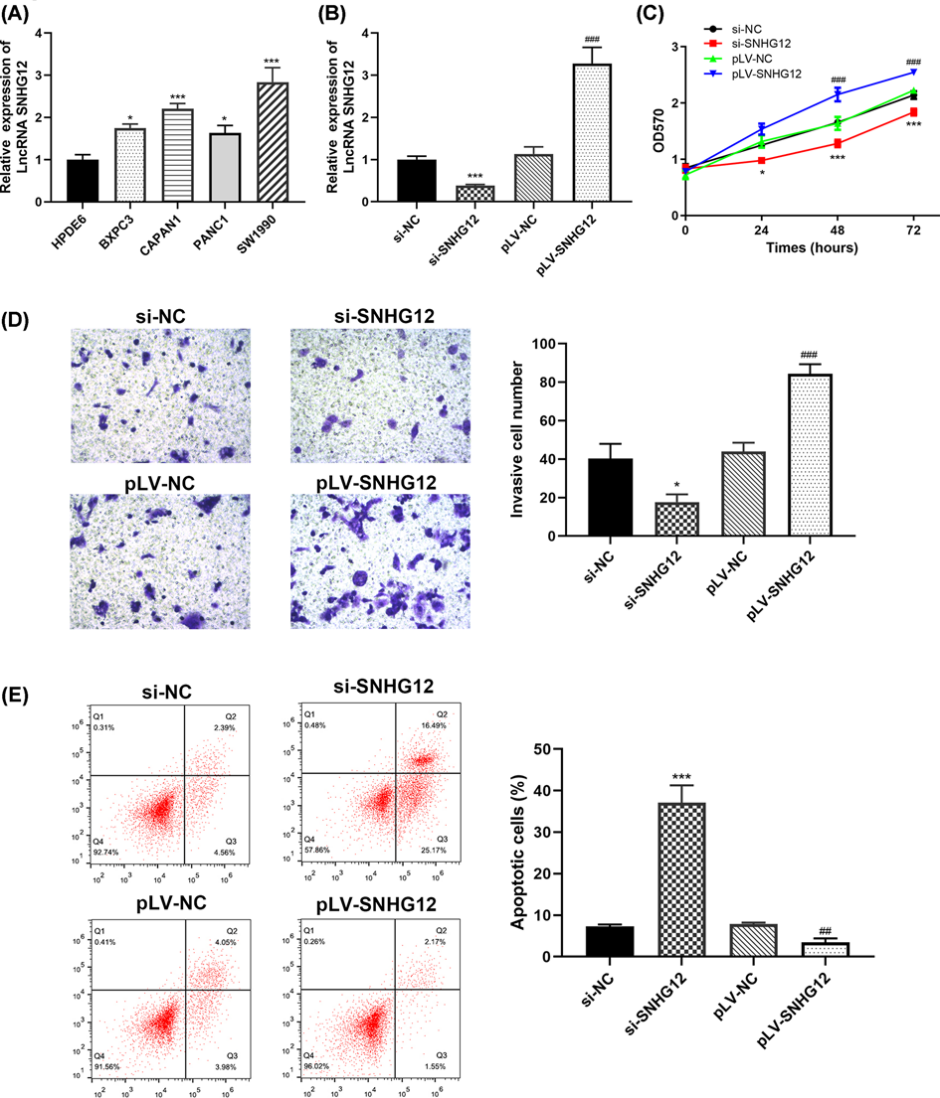
Pancreatic cancer development was proportionate to SNHG12 expression level (**A**) Pancreatic cancer cells had higher SNHG12 up-regulation level than normal pancreatic cells. (**B**) Transfection states of CAPAN1 cells determined by qRT-PCR. (**C**) Results of the MTT assay in CAPAN1 cells with different SNHG12 expression level. (**D**) Results of transwell chamber assay in CAPAN1 cells with different SNHG12 expression level. (**E**) Flow cytometric data of transfected CAPAN1 cells. *P<0.05, ***P<0.001 vs. si-NC, ^##^*P*<0.001, ^###^*P*<0.001 vs. pLV-NC.

The transcription level of SNHG12 was estimated in pancreatic cancer tissues and cell lines. SNHG12 was significantly up-regulated in pancreatic cancer tissues and metastatic pancreatic cancer tissues versus normal tissues and non-metastatic pancreatic cancer tissues (Figure 1A,B), respectively. Four pancreatic cancer cell lines (BXPC3, CAPAN1, PANC1, SW1990) were in higher SNHG12 expression level than normal pancreatic cell (HPDE6) (Figure 2A). The results showed a conspicuous increase of SNHG12 in pancreatic cancer tissues and cell lines.

### Knocking down SNHG12 curtailed pancreatic cancer cells’ capacity of proliferation and invasion, and promoted apoptosis

CAPAN1 cells were selected and cultured for additional exploration of SNHG12 biological function in pancreatic cancer. Related siRNAs (si-NC, si-SNHG12) and pLV-RNAs (pLV-NC, pLV-SNHG12) were transfected into CAPAN1 cells to silence or promote the expression of SNHG12, respectively. Cells were in ideal transfection status after 48 h, which was examined using qRT-PCR (Figure 2A). Immunoblotting test was adapted to reconfirm the transfection states (Figure 2B). MTT assay was performed to estimate the proliferation ability of the pancreatic cancer cells in 72 h. It was unanimous to our hypothesis that the viability of SNHG12 silencing cells escalated at a lower rate than those with pLV-SNHG12 transfection (Figure 2C), which might suggest that SNHG12 was functioned as a promoting factor in pancreatic cancer proliferation. To further investigate the biological function of SNHG12 in cell invasion, we conducted transwell chamber assay and found that invasion rate of pancreatic cancer cells was proportional to the SNHG12 expression level. As showed in the Figure 2D, cells with SNHG12 silencing had significantly less cell invasion than cells transfected with pLV-SNHG12.

Flow cytometry technique was utilized to evaluate the function of SNHG12 in cell apoptosis and cell cycle. As showed in Figure 2E, down-regulated SNHG12 remarkably elevated the apoptosis ratio of pancreatic cancer cells, whereas up-regulated SNHG12 decreased cell apoptosis, compared with cells treated with si-NC.

### Epithelial–mesenchymal transition (EMT) of pancreatic cancer cells was restrained after SNHG12 knockdown

**Figure 3:**
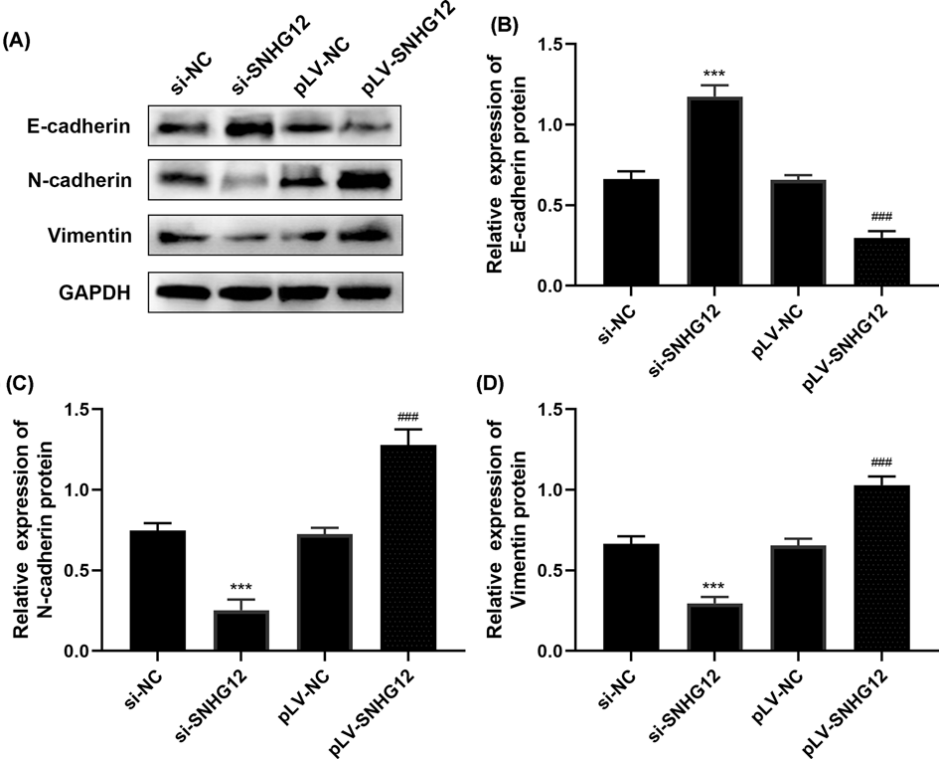
SNHG12 affected EMT progression of CAPAN1 cells by Western blot (**A**) The expression level of EMT-related proteins estimated by immunoblotting assay. (**B–D**) Grey analysis of E-cadherin, N-cadherin and Vimentin proteins using ImageJ. ***P<0.001 vs. si-NC, ^###^*P*<0.001 vs. pLV-NC.

Epithelial–mesenchymal transition (EMT) enable pancreatic cancer converts to a more motile stromal phenotype, which is conducive to the development of peripheral invasion and distant metastasis. It has been confirmed that EMT is an important mechanism involved in the transformation of pancreatitis to pancreatic cancer and metastasis of pancreatic cancer [17,18]. To further explore the mechanism of SNHG12 on invasion, immunoblotting assay was performed to study the EMT-related proteins expression states with E-cadherin, N-cadherin and Vimentin involved. Figure 3 depicted the immunoblotting results of each EMT-related proteins, SNHG12 silencing cells had highest E-cadherin expression level than SNHG12 up-regulated cells. While N-cadherin and Vimentin protein were in the least expression states in si-SNHG12 transfected cells, and were in highest expression level in pLV-SNHG12 transfected cells compared with related si-RNA and pLV-RNA negative controls.

### SNHG12 is of negative regulation on miR-320b

**Figure 4:**
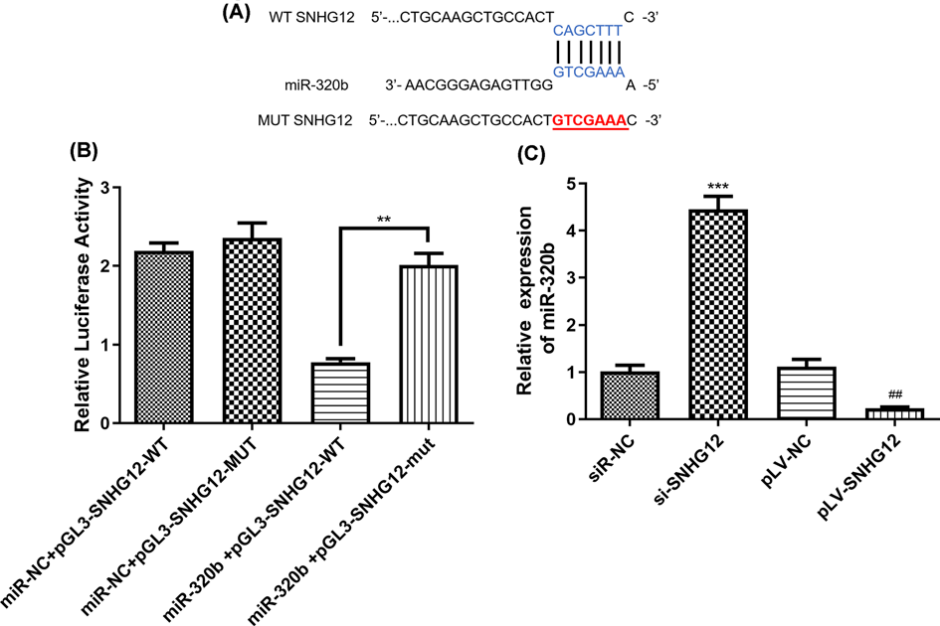
MiR-320b is the target of SNHG12 (**A**) Binding sites of WT SNHG12 and miR-320b predicted by miRanda. (**B**) Results of dual luciferase assay for SNHG12 and miR-320b. (**C**) Expression level of miR-320b in different transfected CAPAN1 cells. WT, wild-type; Mut, mutant. **P<0.01, ***P<0.001, ^##^*P*<0.01 vs. pLV-NC.

Further mechanism of SNHG12 depressing the progression of pancreatic cancer cells was studied and miR-320b was identified as a factor that was negative regulated by SNHG12. The intersection of prediction targets using miRanda, LncBase and Starbase databases showed that there was continuous SNHG12 wild-type binding sites on the miR-320b gene sequence (Figure 4A). We further performed a dual luciferase reporter gene assay to determine whether WT SNHG12 can directly target miR-320b. The results of luciferase activity assay indicated that after co-transfection of a gene plasmid containing the SNHG12 wild -type sequence with miR-320b, luciferase relative activity significantly decreased, which suggested that the expression of SNHG12 in pancreatic cancer cells would be suppressed through the direct binding between miR-320b and WT SNHG12 (Figure 4B).

The expression level of miR-320b was evaluated by qRT-PCR and demonstrated in Figure 4C, the content of miR-320b was in reverse ratio to SNHG12 quantity. SNHG12 up-regulated cells had the least miR-320b within, while the cells with less SNHG12 expressed had the most miR-320b. The results suggested that SNHG12 overexpression reduced mRNA expression of miR-320b in pancreatic cancer cells.

### MiR-320b restored the effects of invasion and EMT triggered by SNHG12

**Figure 5:**
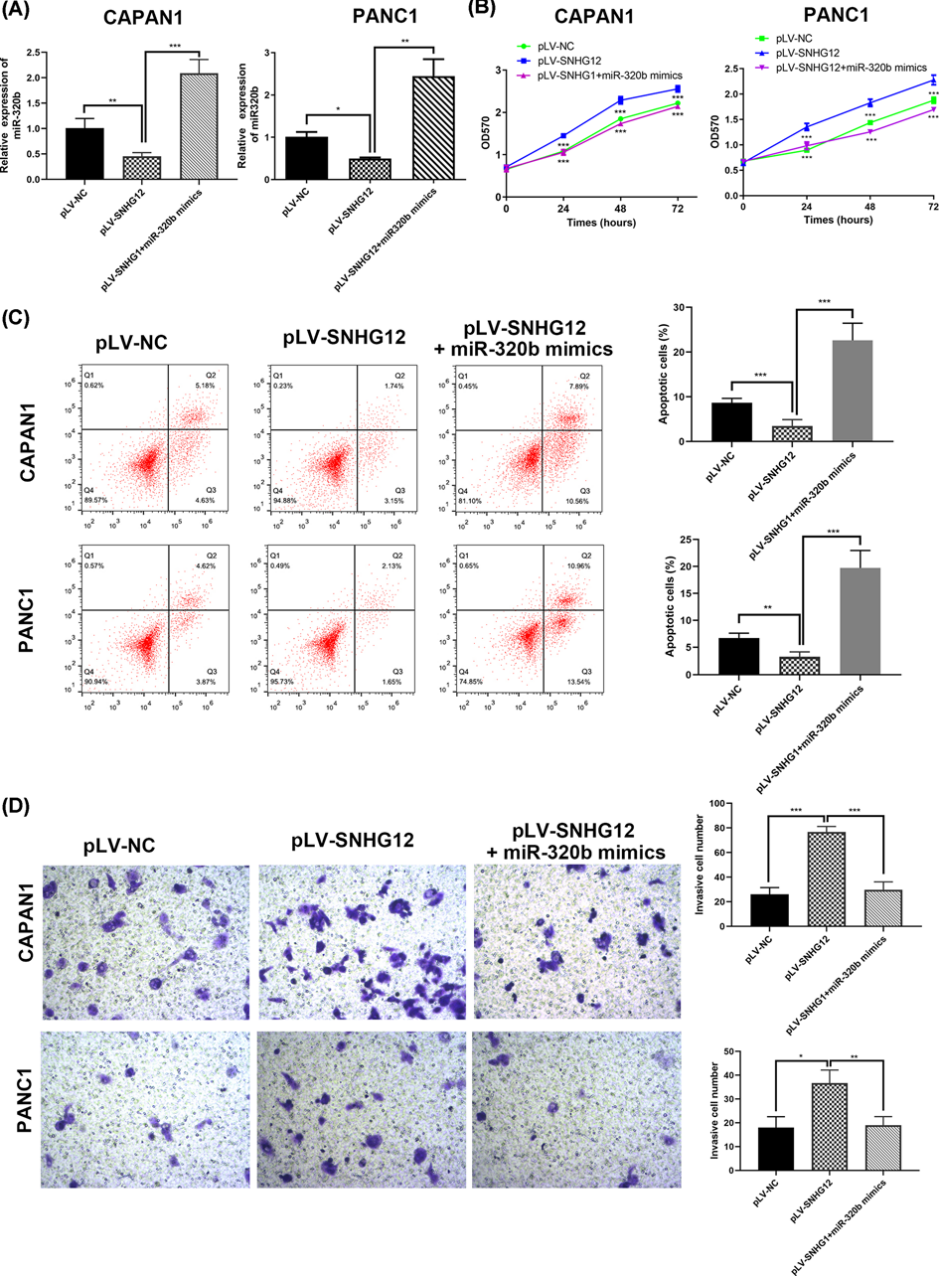
MiR-320b reversed the effects induced by SNHG12 in cell proliferation and invasion in CAPAN1 and PANC1 cells (**A**) miR-320b expression in transfected cells detected by qRT-PCR. (**B**) MTT assay determined the cell growth in different transfected cells. (**C**) Invasion ability evaluated by transwell chamber assay. (**D**) Cell apoptosis rate in transfected cells investigated by flow cytometry. *P<0.05, **P<0.01, ***P<0.001.

In order to figure out whether SNHG12 produces an effect by absorbing miR-320b, two pancreatic cancer cell lines (CAPAN1 and PANC1) with up-regulated SNHG12 were co-transfected with miR-320b mimic (Figure 5A). MTT assay revealed that cell proliferated fastest in the miR-320b group (Figure 5B). Similarly, results of flow cytometric further demonstrated co-transfected miR-320b and pLV-SNHG12 in pancreatic cancer cells showed significantly higher apoptosis rate versus the pure SNHG12 up-regulated cells (Figure 5C).

**Figure 6:**
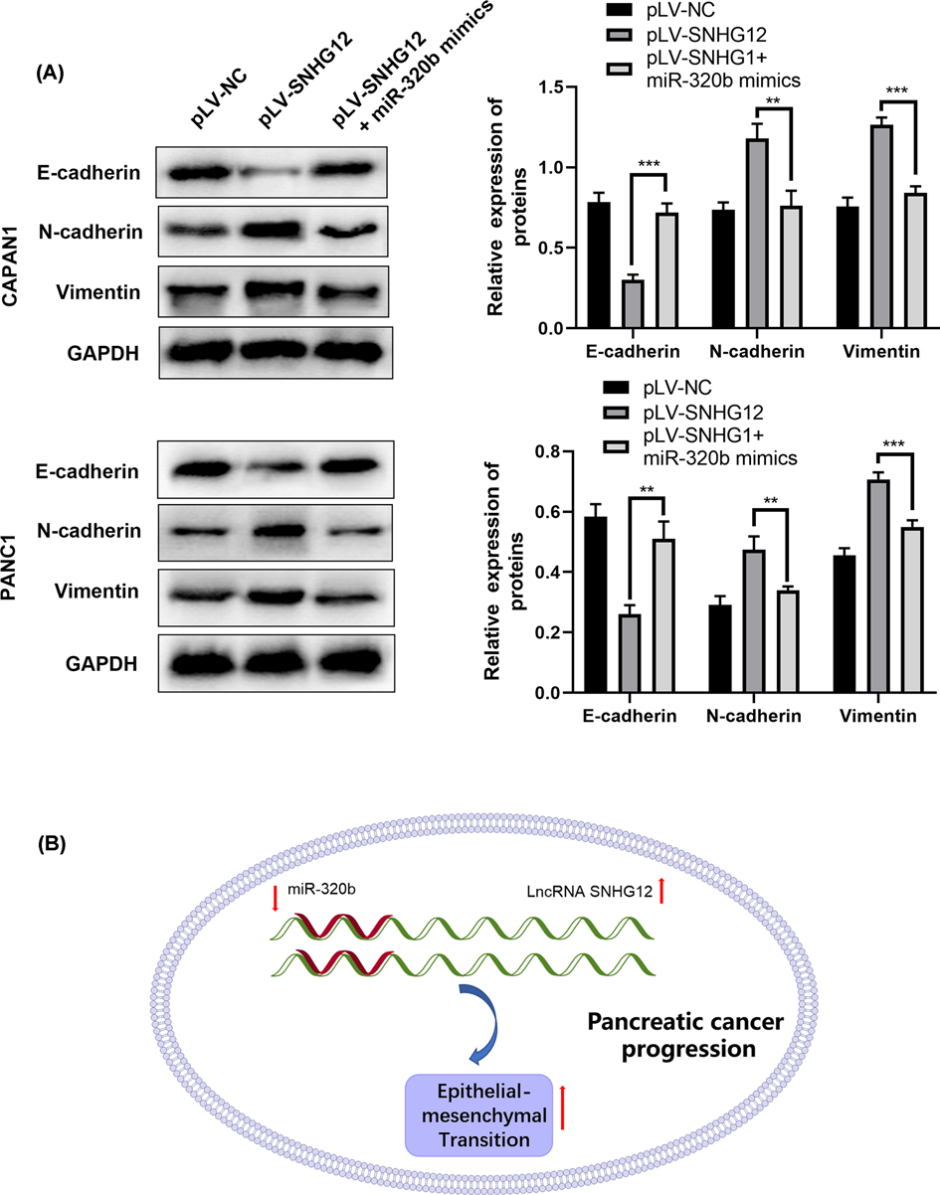
MiR-320b reversed the effects induced by SNHG12 in EMT in CAPAN1 and PANC1 cells (**A**) Results of immunoblotting test in transfected cells, **P<0.01, ***P<0.001. (**B**) Schematic drawing of SNHG12/miR-320b/EMT axis.

Also, the investigation results of capacity and mechanism on cell invasion were consistent with the previous hypothesis that miR-320b repressed pancreatic cancer cells invasion through epithelial–mesenchymal transaction suppression. Transwell chamber assay was conducted to study the difference brought by miR-320b in SNHG12-upregulated cells (Figure 5D), the proportion of cell invasion decreased in miR-320b transfected cells. Next, nuance in miR-320b mimic transfected cells versus the pure SNHG12-upregulated in the epithelial–mesenchymal transaction process of invasion were estimated through Western blotting (Figure 6A). With miR-320b mimic co-transfected with pLV-SNHG12 in pancreatic cancer cells, EMT-related proteins expression was in opposite level in two groups. In miR-320b and pLV-SNHG12 co-transfected group, the content of E-cadherin was obviously higher, while N-cadherin and Vimentin were markedly less than SNHG12-upregulated group, which was consistent with the results in transwell assay.

## Discussion

Although the prognosis of many other malignancies has increased in the past few decades, the overall survival rate of pancreatic cancer has barely improved [19]. Early diagnose of pancreatic cancer is still confronting big challenges because of its insidious symptoms and unobtrusive signs with similarity in mortality and morbidity [20]. Poor prognosis and large probability of postoperative recurrence make 5-year survival rate lower than 20% [21]. Autopsy showed that 90% of pancreatic cancer patients had developed distant metastasis [22]. Although surgery is the predominate method to cure pancreatic cancer, only 15–20% of the patients were respectable in the first diagnosis [23]. Therefore, it is an urgency to find initial diagnostic biomarkers and potential therapeutic targets for pancreatic cancer.

Long non-coding RNA (lncRNA) is a kind of non-coding RNA with tissue and disease specificity [24], which recently had been found to play a crucial role in cell growth, metastasis and invasion of liver cancer, bladder cancer and breast cancer [25–28]. Among them, SNHG12 has been reported in highly expressed quantities in gastric cancer, triple-negative breast cancer and colon cancer with direct proportion to the growth and development of tumor tissue and cells. To the best of our knowledge, however, the function of SNHG12 in pancreatic cancer has not been previously explored. In this research, it is the first time to elaborate the precise pathological relation of SNHG12 and the progression of pancreatic cancer.

Current data in the present study demonstrated that high expression of SNHG12 in pancreatic cancer accelerated the progress of cell growth and invasion. High SNHG12 expression level was first confirmed in pancreatic cancer tissues and cell lines using qRT-PCR, which suggested that SNHG12 could be invoked as a biomarker for early diagnosis of pancreatic cancer. Further investigation on SNHG12 specific function in pancreatic cancer was studied by transfecting corresponding si-RNAs (si-NC, si-SNHG12) into CAPAN1 cells, which identified that knockdown of SNHG12 suppressed pancreatic cancer cells growth by MTT assay. knockdown of SNHG12 inhibited cancer cell invasion that was evaluated by transwell chamber assay. Alleviated apoptosis rate had been verified in SNHG12-downregulated cells. These findings demonstrated that SNHG12 may be involved in the tumorigenesis and progression of pancreatic cancer.

Recent studies on miRNAs have proved that miRNAs have an essential role in the process of tumor development. Zhang et al. found that miR-194 was highly expressed in pancreatic cancer tissues and serum, and overexpression of miR-194 can reduce the expression of DACH1 and promote cell proliferation and migration [29]. Wang et al. showed that miR-320b inhibited cell growth in human colorectal cancer by targeting c-Myc [30]. Similarly, ras-related protein RAP2B was also verified as an interdependent factor of miR-320b to promote the progression of osteosarcoma [31]. Simultaneously, miR-320b was confirmed and validated as a negative regulator of TP53-regulated inhibitor of apoptosis (TRIAP1), and its association with the development of nasopharyngeal carcinoma has been proved [32]. It’s worth noticing that miR-320b was affirmed as a pivotal element in carcinomas that suppressed proliferation, migration and invasion in carcinomas. Here, we identified miR-320b as a negatively regulatory factor of SNHG12. Luciferase activity assay certified that miR-320b is a target of SNHG12 in pancreatic cancer in the present study. Our findings revealed that the function that miR-320b reversed the effects triggered by SNHG12 such as proliferation and metastasis was observed after co-transfection of miR-320b mimic and pLV-SNHG12 to CAPAN1 and PANC1 cells and, which further indicated that the EMT progression was influenced, suggesting SNHG12/miR-320b/EMT axis is possibly a potential target for pancreatic cancer (Figure 6B). The in-depth studies in SNHG12/miR-320b/EMT axis will be explored, including *in vivo* effect and the related other mechanisms.

Consequently, this research elucidated for the first time that SNHG12 is a novel candidate for early diagnosis biomarker and promising treatment target of pancreatic cancer, and SNHG12/miR-320b could be used as alternate therapeutic strategy for better rescuing patients with pancreatic cancer.

## Abbreviations

EMT: epithelial–mesenchymal transition
lncRNA: long non-coding RNA
SNHG12: small nuclear RNA host gene 12

## References

[1] Rahib L., Smith B.D., Aizenberg R., Rosenzweig A.B., Fleshman J.M. and Matrisian L.M. (2014) Projecting cancer incidence and deaths to 2030: the unexpected burden of thyroid, liver, and pancreas cancers in the United States. Cancer Res. 74, 2913–21 10.1158/0008-5472.CAN-14-0155 24840647

[2] Wright G.P., Poruk K.E., Zenati M.S. et al. (2016) Primary tumor resection following favorable response to systemic chemotherapy in stage IV pancreatic adenocarcinoma with synchronous metastases: a bi-institutional analysis. J. Gastrointest. Surg. 20, 1830–5 10.1007/s11605-016-3256-2 27604886

[3] Pereira F., Vasques F., Moricz A., Campos T., Pacheco J.A. and Silva R. (2012) Correlation analysis between post-pancreatoduodenectomy pancreatic fistula and pancreatic histology. Revista do Colegio Brasileiro de Cirurgioes 39, 41–7 10.1590/S0100-69912012000100009 22481705

[4] Jang J.Y., Kang J.S., Han Y. et al. (2017) Long-term outcomes and recurrence patterns of standard versus extended pancreatectomy for pancreatic head cancer: a multicenter prospective randomized controlled study. J. Hepato-Biliary-Pancreatic Sci. 24, 426–33 10.1002/jhbp.465 28514000

[5] Kamisawa T., Wood L.D., Itoi T. and Takaori K. (2016) Pancreatic cancer. Lancet North Am. Ed. 388, 73–85 10.1016/S0140-6736(16)00141-0 26830752

[6] Spizzo R., Almeida M.I., Colombatti A. and Calin G.A. (2012) Long non-coding RNAs and cancer: a new frontier of translational research? Oncogene 31, 4577–87 10.1038/onc.2011.621 22266873 PMC3433647

[7] Flippot R., Beinse G., Boilève A., Vibert J. and Malouf G.G. (2019) Long non-coding RNAs in genitourinary malignancies: a whole new world. Nat. Rev. Urol. 16, 484–504 10.1038/s41585-019-0195-1 31110275

[8] Zhang H., Li W., Gu W., Yan Y., Yao X. and Zheng J. (2019) MALAT1 accelerates the development and progression of renal cell carcinoma by decreasing the expression of miR-203 and promoting the expression of BIRC5. Cell Prolif. 52, e12640 10.1111/cpr.12640 31250518 PMC6797509

[9] Matouk I.J., Halle D., Raveh E., Gilon M., Sorin V. and Hochberg A. (2016) The role of the oncofetal H19 lncRNA in tumor metastasis: orchestrating the EMT-MET decision. Oncotarget 7, 3748 10.18632/oncotarget.6387 26623562 PMC4826167

[10] Shen J., Hong L., Yu D., Cao T., Zhou Z. and He S. (2019) LncRNA XIST promotes pancreatic cancer migration, invasion and EMT by sponging miR-429 to modulate ZEB1 expression. Int. J. Biochem. Cell Biol. 113, 17–26 10.1016/j.biocel.2019.05.021 31163263

[11] Wang O., Yang F., Liu Y. et al. (2017) C-MYC-induced upregulation of lncRNA SNHG12 regulates cell proliferation, apoptosis and migration in triple-negative breast cancer. Am. J. Transl. Res. 9, 533 28337281 PMC5340688

[12] Zhang H. and Lu W. (2018) LncRNA SNHG12 regulates gastric cancer progression by acting as a molecular sponge of miR-320. Mol. Med. Rep. 17, 2743–9 29207106 10.3892/mmr.2017.8143

[13] Song J., Wu X., Ma R., Miao L., Xiong L. and Zhao W. (2019) Long noncoding RNA SNHG12 promotes cell proliferation and activates Wnt/β-catenin signaling in prostate cancer through sponging microRNA-195. J. Cell. Biochem. 120, 13066–75 10.1002/jcb.28578 30945357

[14] Wang J., Xu C., Wu H. and Shen S. (2017) LncRNA SNHG12 promotes cell growth and inhibits cell apoptosis in colorectal cancer cells. Braz. J. Med. Biol. Res. 50, 10.1590/1414-431x20176079 PMC5333723 28225893

[15] Liu X., Zheng J., Xue Y. et al. (2018) Inhibition of TDP43-mediated SNHG12-miR-195-SOX5 feedback loop impeded malignant biological behaviors of glioma cells. Mol. Ther.-Nucleic Acids 10, 142–58 10.1016/j.omtn.2017.12.001 29499929 PMC5751968

[16] Ginzinger D.G. (2002) Gene quantification using real-time quantitative PCR: an emerging technology hits the mainstream. Exp. Hematol. 30, 503–12 10.1016/S0301-472X(02)00806-8 12063017

[17] Zhai L.-L., Wu Y., Cai C.-Y. and Tang Z.-G. (2015) Overexpression of homeobox B-13 correlates with angiogenesis, aberrant expression of EMT markers, aggressive characteristics and poor prognosis in pancreatic carcinoma. Int. J. Clin. Exp. Pathol. 8, 6919 26261579 PMC4525913

[18] Santamaria P.G., Moreno-Bueno G., Portillo F. and Cano A. (2017) EMT: Present and future in clinical oncology. Mol. Oncol. 11, 718–38 10.1002/1878-0261.12091 28590039 PMC5496494

[19] Hahn S., Ayav A. and Lopez A. (2017) Resection of locally advanced pancreatic neoplasms after neoadjuvant chemotherapy with nab-paclitaxel and gemcitabine following FOLFIRINOX failure. Case Rep. Gastroenterol. 11, 422–7 10.1159/000478722 29033758 PMC5624236

[20] Bray F., Ferlay J., Soerjomataram I., Siegel R.L., Torre L. and Jemal A. (2018) GLOBOCAN estimates of incidence and mortality worldwide for 36 cancers in 185 countries. CA Cancer J. Clin. 68, 394–424 10.3322/caac.21492 30207593

[21] Notta F., Hahn S.A. and Real F.X. (2017) A genetic roadmap of pancreatic cancer: still evolving. Gut 66, 2170–8 10.1136/gutjnl-2016-313317 28993418

[22] Frampas E., David A., Regenet N., Touchefeu Y., Meyer J. and Morla O. (2016) Pancreatic carcinoma: key-points from diagnosis to treatment. Diagn. Intervent. Imag. 97, 1207–23 10.1016/j.diii.2016.07.008 27567314

[23] Li X., Guo C., Li Q. et al. (2019) Association of Modified-FOLFIRINOX-Regimen-Based Neoadjuvant Therapy with Outcomes of Locally Advanced Pancreatic Cancer in Chinese Population. Oncologist 24, 301 10.1634/theoncologist.2018-0696 30459238 PMC6519772

[24] Bánfai B., Jia H., Khatun J. et al. (2012) Long noncoding RNAs are rarely translated in two human cell lines. Genome Res. 22, 1646–57 10.1101/gr.134767.111 22955977 PMC3431482

[25] Li H., An J., Wu M. et al. (2015) LncRNA HOTAIR promotes human liver cancer stem cell malignant growth through downregulation of SETD2. Oncotarget 6, 27847 10.18632/oncotarget.4443 26172293 PMC4695030

[26] Wieczorek E. and mRNA R.E. (2018) microRNA and lncRNA as novel bladder tumor markers. Clin. Chim. Acta 477, 141–53 10.1016/j.cca.2017.12.009 29224950

[27] He A., Chen Z., Mei H. and Liu Y. (2016) Decreased expression of LncRNA MIR31HG in human bladder cancer. Cancer Biomark 17, 231–6 10.3233/CBM-160635 27434291 PMC13020480

[28] Augoff K., McCue B., Plow E.F. and Sossey-Alaoui K. (2012) miR-31 and its host gene lncRNA LOC554202 are regulated by promoter hypermethylation in triple-negative breast cancer. Mol. Cancer 11, 5 10.1186/1476-4598-11-5 22289355 PMC3298503

[29] Zhang J., Zhao C.-Y., Zhang S.-H. et al. (2014) Upregulation of miR-194 contributes to tumor growth and progression in pancreatic ductal adenocarcinoma. Oncol. Rep. 31, 1157–64 10.3892/or.2013.2960 24398877

[30] Wang H., Cao F., Li X. et al. (2015) miR-320b suppresses cell proliferation by targeting c-Myc in human colorectal cancer cells. BMC Cancer 15, 748 10.1186/s12885-015-1728-5 26487644 PMC4617986

[31] Yang C., Wu K., Wang S. and Wei G. (2018) Long non-coding RNA XIST promotes osteosarcoma progression by targeting YAP via miR-195-5p. J. Cell. Biochem. 119, 5646–56 10.1002/jcb.26743 29384226

[32] Li Y., Tang X., He Q. et al. (2016) Overexpression of mitochondria mediator gene TRIAP1 by miR-320b loss is associated with progression in nasopharyngeal carcinoma. PLoS Genet. 12, e1006183 10.1371/journal.pgen.1006183 27428374 PMC4948882

